# Exploring the Role and Functionality of Ingredients in Plant-Based Meat Analogue Burgers: A Comprehensive Review

**DOI:** 10.3390/foods13081258

**Published:** 2024-04-19

**Authors:** Gil Vila-Clarà, Anna Vila-Martí, Laia Vergés-Canet, Miriam Torres-Moreno

**Affiliations:** 1 Research Group M3O, Methodology, Methods, Models and Outcomes of Health and Social Sciences, Faculty of Health Sciences and Welfare, University of Vic—Central University of Catalonia, 08500 Vic, Spain; gil.vila@uvic.cat (G.V.-C.); miriam.torres@uvic.cat (M.T.-M.); 2 Zyrcular Protein Labs, SL2, 28001 Madrid, Spain; lverges@zyrcularfoods.com; 3 Institute for Research and Innovation in Life Sciences and Health in Central Catalonia (IRIS-CC), 08500 Vic, Spain

**Keywords:** meat analogues, plant-based meat, plant-based burgers, functionality, food technology innovation, clean-label ingredients, composition, sustainability

## Abstract

The development of plant-based meat analogues has become a significant challenge for the food industry in recent years due to the increasing demand for sustainable and healthier proteins in the context of a global protein transition. Plant-based meat analogues imitate the visual, textural, and chemical properties of traditional meat products and are required to closely resemble meat to appeal to consumers. In addition, consumers demand natural, clean-label, and nutritional, and healthy products. To address these challenges, the food industry must develop highly healthy, nutritious, and E-number-free meat analogue products. Understanding the functionality of each ingredient and its role in the food matrix is crucial to being a key player in the innovation of the meat analogue market. This review provides updated information on the primary ingredients utilized for the development of plant-based burger meat alternatives and their functionality. The key components of meat analogue burgers are outlined, including plant proteins, binding agents, fats and oils, flavorings, colorings, preservatives, fortificants, and clean-label considerations.

## 1. Introduction

Meat, as a protein source, has been widely consumed by humans since the prehistoric era due to its energy contribution, its contribution of high-quality proteins for the construction of body structures such as muscle, its palatability, as well as its association with elements such as strength or power. Nowadays, most omnivorous populations consume meat for different reasons, and those related to hedonism and dependence as a habit stand out. People who have a greater affinity towards meat show negative attitudes towards reducing their consumption or eating a plant-based diet being more likely to eat meat more frequently and less likely to make changes to their diet [1].

The ever-increasing human population and food habits have increased the consumption of meat across the globe, with an expected growth of more than 70% in the demand for meat by 2030 [2].

This massive demand for animal protein, especially in the form of meat, expected in the coming years due to the climate emergency has created the need to look for alternative protein sources since the production of animal protein has a high impact at an environmental level [3,4,5,6]. This explains there is currently a high global interest in plant-based ingredients and foods, as well as introducing new ingredients, such as new sources of protein for human nutrition that are healthier and, at the same time, with less environmental impact than proteins of animal origin, especially those obtained through meat, which also respond to concerns like an increasing consumer awareness of more environmentally responsible consumption. This concern is not only attributable to the population that follows a vegetarian or vegan diet, but also to those omnivorous consumers who want to reduce the consumption of meat in their diet and who can incorporate alternative products in their diet as a healthy alternative and as part of a more environmentally friendly diet. In this sense, different studies have concluded that the population that usually eats meat might be willing to switch to alternative meat products as long as they have organoleptic properties similar to meat [7]. This is the main reason why the food industry develops products like meat but of plant origin, which are called plant-based meat analogues (PBMAs).

The topic of PBMAs has become one of the biggest challenges for the food industry in recent years. Meat analogues are categorized as food products that imitate the visual, textural, and chemical properties of traditional meat products, including their odor, flavor, taste, juiciness, and mouthfeel [8]. When developing these products, it is essential to consider crucial factors, such as the digestibility and bioavailability of nutrients, ensuring that PBMAs are easily digestible and offer optimal nutrient absorption. Therefore, it is necessary that these closely resemble meat in order to appeal to consumers and encourage them to modify their dietary habits by reducing their meat intake [9]. Additionally, the price and shelf-life of PBMAs are important factors that influence consumer purchasing decisions [7]. Furthermore, due to the increase in global awareness of environmental concerns, factors such as sustainable ingredient sourcing and packaging have a significant impact on the acceptance of a product.

Lastly, as consumers become increasingly interested in making healthier food choices, there is an increasing demand for natural, clean-label, and nutritional products. Thus, the food industry faces the additional challenge of developing PBMAs that are both highly nutritious and free of E-numbers. So, in order to be a key player in the innovation of the meat analogue market, it is indispensable to know which product types exist and their respective technological process. Additionally, understanding the functionality of each ingredient and its role in the food matrix is essential.

The renewed devotion of global food scientists to alternative studies in the past few years has led to a spike in research publications on meat alternatives [10]. As of today, there is a lot of published knowledge detailing relevant information on PBMAs in different areas. Research regarding consumer preferences and attitudes has given an overview of how PBMAs are perceived and accepted by consumers. Fiorentini et al. [8] described the role of sensory evaluation in the consumer acceptance of meat analogs. On the other hand, Boukid [11] and He et al. [12] depicted in their article the key motivators and demotivators behind consumer’s purchasing/consuming plant-based meat analogues. In this area, other authors studied the consumer perception of meat analogues, such as Slade [7], who evaluated the willingness to purchase plant-based burgers; Wild et al. [13] investigated consumers’ motivation to shift their diets toward meat alternatives, Aschemann-Witzel and Peschel [14] explored the ingredient perception of PBMAs and Moussaoui et al. [15] studied consumers’ responses to PBMA burgers according to their attitude towards meat reduction. The information regarding nutritional composition has been studied from different perspectives. Some authors have assessed the nutritional profile of PBMA products from different areas, such as Rizzolo-Brime et al. [16] and Costa-Catala et al. [17] for Spain, Bryngelsson et al. [18] for Sweden, Cutroneo et al. [19] for Italy and Boukid and Castellari [20] and Bohrer [21] for a global market perspective. These analyses provide a more comprehensive understanding of the nutritional profile compared to meat products. In the production area, other authors have contributed to the expansion of knowledge on the processing technologies behind meat analogue products (traditional and new emerging technologies), such as Dekkers et al. [22], Plattner [23], and Kyriakopoulou et al. [24] who reviewed the different structure processing technologies for meat analogues or Fu et al. [25] and Mosibo et al. [26] who gave better insights of the potentials and challenges of processing novel ingredients. Other studies have been focused on describing the formulation and the main ingredients present in PBMAs. Some examples are Kołodziejczak et al. [27], Sha and Xiong [10], and Ishaq et al. [28], who described the ingredient composition of PBMAs or Kyriakopoulou et al. [29], who classified PBMAs in different categories and elucidated the functionality of their ingredients. Table 1 depicts the different articles that have described the different ingredients of PBMA products. However, despite the abundance of studies in the literature on the general ingredients of meat analogues, there remains a noticeable gap in articles focusing exclusively on the detailed functionalities and roles of the ingredients found in PBMA burgers.

Hence, this review aims to address this gap by examining the available scientific literature and providing updated insights into the primary ingredients used in the development of plant-based burger meat alternatives, along with their functionality.

## 2. Composition of Meat Analogues

Tofu, tempeh, and seitan are plant-based products with some relative characteristics for meat. For some parts of the population, these products are considered meat alternatives even though they are not traditionally viewed as meat substitutes in their countries of origin. These products are often referred to as the first generation of PBMAs, while newer products based on texturized vegetable proteins (TVPs) are described as the second generation of PBMA [12,22].

Meat analogues can be classified as plant-based (soy, pea, gluten, etc.), cell-based (cultured meat), fermentation-based (mycoproteins), insect protein-based, and microalgae proteins [10,30].

Plant-based ingredients can be used to create a variety of PBMA products. Some examples include burgers, sausages, pâté, meatballs, nuggets, fish, seafood, bacon, chicken chunks, minced meat, pulled meat, and more. Several studies have established different categories for classifying these products [16,27,28,29]. The sensory and textural characteristics of PBMAs are primarily determined by the selection of ingredients, and these alternatives typically contain 50–80% water and include non-textured-based proteins, vegetable textured-based proteins, fats, additives to such flavorings, coloring agents, and binding agents [30].

Water is an important ingredient in meat alternatives for its multiple functions during processing and within the food matrix of the final product, reducing costs. Proteins, together with water, are the main components of many PBMAs, and these are responsible for providing texture, taste, and physical appearance. Vegetable proteins have intrinsic physicochemical and functional properties that directly impact the characteristics of the final product [33], and these properties tend to vary depending on how the proteins are processed. Regarding lipids, the total fat content of PBMAs is generally similar to that of traditional meat products, although the fatty acid profile is different [34] due to the variety of fatty ingredients, fats, and oils that are used in the formulation [20,21]. When analyzing meat products, carbohydrates are generally not found in meat unless the product is further processed. In contrast, PBMAs typically contain significant amounts of carbohydrates, and these provide texture, consistency, binding capacity, and product stability and have a direct impact on the nutritional profile. In this case, depending on the ingredients, fiber content is significant, as a feature not typically observed in animal burgers [35]. While this is true for most PBMA products, there are some exceptions, such as some chicken chunks or strips that are basically made with protein and fat ingredients [21]. In prepared meat products that are minimally or further processed, culinary spices, herbs, fruits, seeds, essential oils, and different plant extracts are extensively used to modify product flavor. The same happens in PBMAs, where seasonings and spices, as well as various meaty flavorings, are added to mimic the flavors and aromas of meat [36]. Normally, colorings are included as artificial or natural colorants to achieve the desired appearance. Furthermore, some PBMAs are fortified with essential vitamins and minerals that are typically found in meat, such as vitamin B12 or iron. This helps to address potential nutritional deficiencies in vegan or vegetarian diets and increase the overall nutritional value of the product.

## 3. Plant-Based Burgers: Ingredients and Functionality

The meat analogue market is expanding very fast (with profits of USD 6.1 billion in global sales in 2022), and hundreds of innovative products are being released [37]. Currently, burgers are the primary product of this new generation of PBMA, along with other products, such as ground beef, sausages, bacon, and hotdogs [12]. In this paper, only PBMA burgers based on TVP are considered.

Plant-based burgers resembling animal-based burgers aim at recreating their appearance, odor, flavor, distinct bite, chewiness, succulence, and firmness, among other attributes. Animal-based burgers consist mainly of proteins and fats and, to a lesser extent, seasoning, salt, and binders. Plant-based burgers follow relatively the same recipe; however, the nutritional composition can differ substantially [16,17,20,34]. The principal constituents of plant-based burgers are texturized vegetable proteins and, to a lesser extent, flours, protein concentrates or isolates, fats, and other minor components such as binders, seasonings, salt, etc. [28]. Table 2 provides an overview of the source and primary functionality of various ingredients categorized by their macronutrient composition in plant-based meat analogue burger formulation. In the following sections, these ingredients are explained in more detail, attending to the functionality of each ingredient and its role in the food matrix.

### 3.1. Plant Proteins

The protein ingredient type (pea protein, soy protein, wheat protein, etc.) used in the formulation of plant-based burgers is one of the main components for product identity and product differentiation [21]. The selection of plant proteins as a raw material for the formulation of PBMAs is strongly influenced by cost and technological properties.

The protein functionality includes solubility, emulsification, foaming, gelation, water- and oil-holding capacities, flavor binding, texturization, dough formation, etc. These functionalities are dependent on the protein composition (chemical composition, amino acid sequence, secondary and higher order structure) and environmental factors (pH, ionic strength, temperature, etc.) [30]. These two parameters are essential in the structure formation of PBMA burgers [24]. Considering these parameters, building proteins soy, pea, and wheat proteins are the most widely used in the formulation of PBMA burgers [10,27]. However, plant proteins from other sources are also used. Some examples are oilseeds (rapeseed, canola, sunflower, etc.), cereals (rice, barley, etc.), or legumes (lentils, lupine, chickpea, etc.) [28]. Plant-based burger manufacturers can incorporate proteins into their products, such as flour, isolates, protein concentrate, or texturized proteins [41]. However, the principal constituents tend to be protein in its texturized form with the aim of mimicking the fibrillar structure of meat muscle [30].

There are several methods available for texturizing vegetable proteins, including wet spinning, electrospinning, shear cell technology, and extrusion. Of these methods, extrusion is the most used technique [22]. Within this technique, two main types of extrusion processes exist based on the amount of water used: low moisture extrusion (<35% moisture content) and high moisture extrusion (40–80% moisture content) [11].

**Figure 1:**
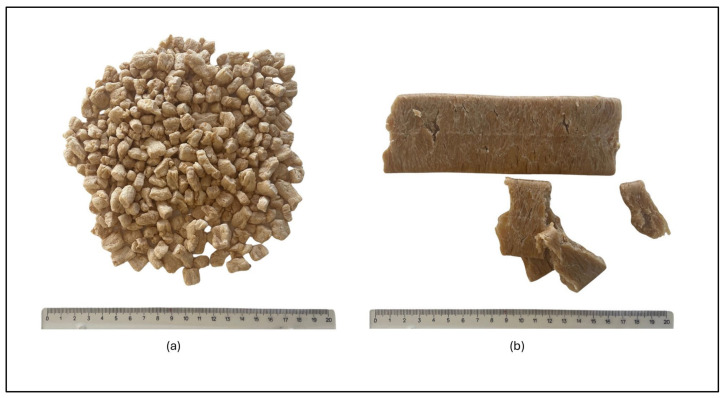
(**a**) Pea protein extrudates (TVPs) obtained by low moisture extrusion and (**b**) Faba bean and pea extrudates (HMMAs) obtained by high moisture extrusion.

Low-moisture extrusion produces a dry texturized vegetable protein (TVP) (Figure 1a), which is used in the formulation of PBMA burgers but needs to be rehydrated. Through the rehydration step, the TVP absorbs water rapidly and obtains a fibrous and spongy nature resembling meat particles [10]. Incorporating hydrated TVP into product formulation can provide the desired meaty and chewy texture while also imparting desirable juiciness [24]. Consequently, TVPs with different granulometries and shapes, such as in chunk form, minced form, and flaked form, are available on the market as food ingredients specifically designed for the formulation of PBMA burgers [26,42]. The selection and combination of these ingredients play a crucial role in achieving the desired texture of the final product. Additionally, a grinding step may be performed after rehydrating certain TVPs to achieve a specific texture.

High moisture extrusion (HME), on the other hand, produces fibrous extrudates and high moisture meat analogues (HMMAs) (Figure 1b). These are high in water content and are primarily suitable for whole-muscle meat analogues but also for other products such as PBMA burgers. Therefore, these HMMAs can be used as an ingredient alone or in combination with rehydrated TVP [43].

Overall, low moisture TVP is more commonly used in the industry due to its lower cost in large volumes, greater flexibility in creating textures, and longer shelf life compared to HMMA [23,44]. The most common extruded ingredients incorporated in PBMA burgers are soy, wheat, and peas. However, other plant proteins such as cottonseed, rapeseed, peanuts, and sesame seeds have also been investigated [25,42].

The use of soybean is worldwide, and many of the PBMAs commercialized are based on soy protein due to its availability, specific properties, and low prices [32]. The functional properties of soy proteins, including their ability to hold water, form gels, absorb fat, and emulsify, make them a popular choice for use in meat analogues. These properties enable the creation of products that closely mimic the texture and mouthfeel of meat. Moreover, from a nutritional point of view, processed soy protein has a very balanced amino acid composition and a high Protein Digestibility Corrected Amino Acid Score is mostly identical to the animal protein [32]. Textured soy proteins, when hydrated, can emulate meat-like textures and are, therefore, highly versatile food ingredients [45]. Non-textured soy protein concentrates and isolates can also be used in PBMAs to boost protein content and enhance other desirable qualities, such as fat and water retention, emulsification, and nutritional composition, ultimately contributing to mouthfeel and texture [42,45]. Compared to unprocessed or minimally processed soy proteins, soy protein concentrates and isolates are preferred due to improvements in color and flavor since minimally processed soy proteins are typically darker and more bitter in taste [21].

As mentioned, wheat protein is also widely used, often in combination with other proteins, specifically gluten protein. Gluten is a structural protein naturally found in certain cereal grains, which is cost-effective and a widely used ingredient in the formulation of PBMA burgers [20]. Wheat gluten is valued for its ability to form a cohesive viscoelastic network through intramolecular and intermolecular disulphide bonds, which is one of its primary functionalities [46]. By imparting a fibrous and compact structure to extrudates, wheat gluten is a crucial ingredient in achieving a meat-like texture that closely resembles real muscle meat [47]. This ingredient plays a crucial role in PBMA burgers as it serves as both a binder and structuring agent. Its unique functionality allows it to create a network that binds the TVP and other ingredients together, resulting in a uniform textured burger [29]. While gluten remains one of the most commonly declared allergens in PBMA burgers, there is a growing demand for allergen-free options [20]. As such, companies are investing in the development of gluten-free burgers that offer a promising texture. However, creating a gluten-free burger with a texture that is similar to traditional burgers remains a significant challenge.

Similar to soy, pea proteins can be used in the formulation of PBMA burgers as TVPs, HMMAs, flour, concentrates, or isolates. Since they can be added in different forms, the functionality differs [29]. While soy protein-based products are the most common meat alternatives, pea protein-based products are gaining in popularity. This is due to the fact that a significant number of customers experience food intolerances from soy and wheat products [48]. Also, pea protein is considered a more sustainable protein in comparison to soy; however, it also has some disadvantages, such as its price, limited amino acid profile, and digestibility. Moreover, depending on how the protein is processed, peas can have a stronger off-flavor that could negatively affect the acceptance of the product [49].

Previously, soybean and wheat gluten were the primary raw materials used in food production; however, with advances in technology and innovation, a wide range of alternatives are now available. These include rice, potato, corn, barley, oats, sorghum, beans, lentils, peas, lupines, and many more, which have already offered promising results [48]. To meet the growing demand for plant-based protein sources, the industry is continuously innovating and diversifying protein portfolios. This has led to the emergence of new protein sources such as fava bean protein, mug bean protein, microalgae, seaweed, fungi, and sweet lupin [11].

### 3.2. Binding and Texturizing Agents

To mimic meat properties, PBMA relies on various ingredients that serve as stabilizers, gelling agents, thickeners, and emulsifiers. In PBMA burgers, binding agents derived from plants are used to bind water and fat and provide adhesion for the TVP particles. Depending on their quantities, some ingredients can act as both binders and extenders [24]. For example, protein ingredients, such as soy protein isolate, pea protein isolate, or wheat gluten, are particularly effective as binding agents due to their water binding and protein network formation capacities.

Carbohydrates are commonly used for their thickening and emulsifying properties, particularly in improving texture consistency, binding water, and reducing syneresis [10]. In fact, these polymers can play a crucial functional and structural role in shaping PBMA burgers [11], and some examples are crude fibers of plant cell wall material, digestible starches, and purified polysaccharides and derivates. Flours and starches, such as those derived from potato, corn, wheat, cassava, pea, and rice, are commonly used as fillers to improve the texture and consistency of PBMA burgers, and maltodextrins or dextrose might be used with the same purpose. In addition, fibers from various sources, including peas, potato, oats, soybean, bamboo, konjac, citrus, and apple, as well as polysaccharide gums like locust bean gum, acacia gum, tara gum, guar gum, xanthan gum, and carrageenan, can act as binding agents to enhance product stability, thickness, and consistency, and reduce cooking loss [10,11]. In this line of research, multiple ingredients and combinations exist.

Fat is not classified as a binder or texturizing agent; however, it is a crucial ingredient when it comes to the textural properties of PBMA burgers since extrudates require a source of fat and binders. The combination of a protein-rich extrudate matrix, binding agents, and fat source helps to retain moisture during post-production frying. This combination is necessary to replicate the juiciness of a meat burger patty and enhance the overall acceptability of the product.

Some other ingredients used as binders or texturizers are methylcellulose (MC), hydroxypropyl methylcellulose, long-fiber cellulose, corn zein, and alginates. It is well documented that these ingredients, besides having a water- and fat-binding capacity, can significantly improve the texture and appearance of the burger by binding texturized vegetable proteins, enhancing oil encapsulation, and reducing oil absorption [29]. MC, in particular, is a dietary fiber that is widely used in PBMA burgers and other types of meat analogues due to its remarkable binding capacity. When added in appropriate amounts to food products, this ingredient can undergo unique reversible thermal gelation and control ice crystal formation, resulting in reduced cooking loss [29,32].

Transglutaminase is an enzyme that can effectively bind protein molecules in plant-based meat analogue products by inducing crosslinks and building up polypeptides. When used in the right amounts, this enzyme can improve the hardness of PBMA burgers by creating fibrous structures [41,50]. It is important to note that this enzyme needs to be declared in the ingredient list in case it is not destroyed during the process, which might result in its rejection by consumers [51].

Lastly, salt addition to the protein base of the PBMAs can lead to the solubilization and unfolding of the protein, affecting its functional properties. For instance, its structuring potential can be enhanced by modifying protein–hydrogen interactions [29,30].

### 3.3. Fats and Oils

In PBMA burgers, the addition of vegetable fat into the formulation can provide similar functionalities to the fat present in meat products. Fat plays a crucial role in the nutritional value and sensory properties of meat and its alternatives, including tenderness, mouthfeel, juiciness, and flavor release [52]. To replicate the flavor and aroma typically associated with meat, it is crucial to carefully consider both the source and the fatty acid profile of the fat used [11].

Vegetable fats do have a disadvantage in that they lack the meat-specific volatile substances found in animal fat. However, may offer health benefits due to their lack of cholesterol and lower saturated fatty acid profile.

When comparing PBMA burgers with traditional meat burgers, it is important to note that while the lipid content is roughly equivalent, the fatty acid profile differs substantially [17,21]. Plant-based burgers tend to be higher in polyunsaturated and monounsaturated fatty acids, in particular linoleic acid (C18:2n6), probably due to the addition of crop seeds and vegetable oils such as sunflower oils [12,34].

The range of different oils varies from product to product, where most PBMA burger recipes include vegetable oils with low amounts of saturated fatty acids (SFAs), although some formulations may contain vegetable oils or fats that are high in SFA [20]. Commonly used oils low in SFA are sunflower oil, olive oil, corn oil, turnip oil, and canola (rapeseed) oil, whereas oils high in SFA are coconut oil, palm oil, and cocoa butter [16,20,34]. The fatty acid composition of fats and oil varies between sources and manufacturing methods, and these determine its physicochemical characteristics and functional properties [21]. The melting point of oils is directly influenced by their fatty acid profile, with oils high in saturated fatty acids (SFAs) generally exhibiting a higher melting point compared to oils low in SFA content. This means that, depending on the oil source and processing method, fats can exist within the food matrix in different forms: liquid, solid, emulsified, crystallized, etc. So, in some cases, in order to develop the texture and mouthfeel resembling animal fat, solid fats are blended with liquid oils that contain more unsaturated fatty acids. Technologies such as encapsulation, emulsion, and oleogelation can be utilized to minimize the separation of plant oils from the product. These methods protect the oils, causing them to release slowly during cooking and consumption [37].

Therefore, the application of various forms of lipids in the proper balance is of high importance since it largely influences the structure, rheological properties, and sensory characteristics [53]. The fat of meat burgers tends to be solid at room temperature and melt upon heating. Hence, an ideal PBMA burger should replicate this property by giving a pleasant mouthfeel similar to the corresponding meat [29]. Moreover, it is important to consider additional parameters, such as the distribution of fat within the food matrix, as this can significantly impact the sample’s hardness and, in consequence, the overall acceptability of the product [54].

### 3.4. Flavoring Agents (Taste and Flavoring Enhancers)

When making purchase decisions, taste is a crucial factor alongside price, healthfulness, convenience, and environmental sustainability. However, optimizing the flavor and taste of PBMA burgers can be challenging because it heavily relies on the raw materials used in their formulation [49].

Soy and legume ingredients are known to have unpleasant flavor profiles and intrinsic off-flavors that can negatively affect the acceptability of PBMA products [55]. Furthermore, plant protein concentrates and hydrolysates are associated with “green”, “grassy”, or “beany” off-odors, as well as long-lasting bitter and/or astringent off-tastes [49]. In soy or peas, off-notes can arise due to the oxidation of unsaturated fatty acids and the presence of glycosides like saponins and phenols such as isoflavones, catechins, and phenolic acids [56]. To overcome these off-flavors and aromas in plant proteins, various processing strategies have been implemented, including purification, fermentation, defatting, and the removal or deactivation of lipoxygenases, among others [49]. However, it remains a challenge for the industry to completely eliminate problematic off-flavors rather than merely masking them.

In PBMA products, a higher quantity of flavor compounds is typically utilized compared to meat-based products. This is because these compounds not only serve to replicate the aroma and taste of meat but also mask any unpleasant aftertaste from the use of specific raw materials [27]. To make PBMA burgers appealing to consumers, their flavor should closely resemble that of traditional meat burgers to mimic beef, lamb, pork, turkey, duck, deer, yak, bison, or other desirable meat flavors [57]. Additionally, the flavor profile of PBMA burgers should be adapted to the geographic zone of distribution, as flavor preferences can vary across regions. To achieve this, various flavors have been patented to offer a wide range of meaty flavors and aromas, such as beefy, bacon-like, umami, savory, bloody, brothy, gravy, metallic, and bouillon-like. Some of these meat-like flavors are developed using different precursors, such as reducing sugars, amino acids, nucleotides, vitamins, and iron complexes [57].

Also, flavoring ingredients can be of natural origin and be labeled as natural flavorings. These include the use of savory yeast extracts, spices, and herbs, such as oregano, sage, black pepper, paprika, rosemary, cloves, and many others [10,11]. Furthermore, during the cooking process, the Maillard reaction occurs, generating new flavor substances from reducing sugars and amino acids. Among the resulting aromas, roast and meaty aromas are the most desired [38,58]. On the other hand, to prevent oxidative reactions and rancidity, antioxidants can be added to PBMA burgers. Organic acids or phosphate compounds can also be used to enhance stability and shelf life and modify the final flavor of the product. Additionally, salt is essential for taste perception and can contribute to extending the product’s shelf life and enhancing its texture. This could explain why PBMA burgers, in some cases, have a higher salt content compared to animal-based burger products [17,20].

### 3.5. Coloring Agents

Color is an important attribute that contributes to the overall product acceptance by consumers [59], and it is particularly significant in meat products, where, in some cases, it is considered to be one of the most important quality characteristics [30].

The same as flavor, there is not a general rule of color attributes for PBMA burgers. These characteristics are mainly determined by the type of burger being simulated. A wide variety of burgers mimicking different animal products exist; some examples are beef burgers, chicken burgers, cod burgers, hake burgers, etc. So, a wide variety of ingredients are used as coloring agents in PBMA burgers, and these vary from product to product.

The raw material ingredients typically used for PBMAs, such as soy protein or gluten, have a beige or yellow-brown color, which is far different from the well-known meat burger appearance. Therefore, colorants can be added either before the extrusion process or during the final product formulation stage, along with the other ingredients [60]. Furthermore, the importance of color changes during preparation cannot be overstated, as these are critical in making PBMA burgers resemble traditional meat products. PBMA burgers are meant to replace raw meat products, which means that they must replicate the typical color changes that occur during cooking [21]. To achieve a meat-like appearance, reducing sugars and heat-stable coloring agents are combined with heat-labile colorants that allow for a color change similar to that of meat during cooking [29].

For colors resembling raw uncooked ground meat, mainly beetroot derivates are being used, such as beetroot juice, powder, concentrate, extract, etc. For colors resembling brown or cooked meat, caramel, annatto, turmeric, and other artificial dyes are being utilized [39,61,62]. The choice of pigments and their amount in a coloring composition can vary based on the desired color of the ground meat.

Also, it is important to note that during cooking, the Maillard reaction takes place, which can lead to desired color changes. To enhance their reaction, various combinations of reducing sugars such as dextrose, maltose, lactose, xylose, galactose, mannose, and arabinose have been proposed in different formulations [38,39]. Additionally, the apple extract has been suggested to enhance color changes due to the oxidation of polyphenols and ascorbic acid during cooking [10].

In addition to the additives mentioned earlier, pigment extracts from red cabbage, red berries, paprika, and carrots may also be used in PBMA burger formulations to achieve desired colors. Some of these ingredients and others might be of high interest since they can be perceived as natural or can be labeled in the ingredient list as spices or spice extracts. Also, in some PBMA burgers, the use of soy leghemoglobin has been used to create an aroma and a “bloody” appearance of meat heme proteins like hemoglobin and myoglobin [63,64].

These additives are employed to keep the product’s intended color by inhibiting the color from bleeding out while it is being processed or stored [65]. Moreover, it is important to note that the pH level of PBMA burger formulations can have a negative impact on the stability of the colorants used. To address this issue, acidulants such as citric acid, acetic acid, lactic acid, or their combinations can be added to achieve an optimal pH range. In some cases, maltodextrin and hydrated alginate can also be used to inhibit or control color migration from the dyed structured PBMA and preserve color retention [65].

### 3.6. Preservatives

Maintaining a long shelf-life is an important aspect of PBMA production, as consumers expect their food products to remain fresh and safe for consumption for extended periods of time. Therefore, it is important to control and prevent the growth of microorganisms in PBMA burgers to extend their shelf life [48]. Table 3 highlights different methodologies to extend the shelf-life of PBMA burgers, which are discussed in the following paragraphs.

At the moment, there is limited information available regarding the food safety risks associated with PBMAs; however, it is important to note that due to their high protein and moisture content, as well as almost neutral pH, these products are susceptible to spoilage [13]. Nowadays, a variety of preservation methods are available that can help extend the shelf life of food products. These methods often involve the use of food preservatives, which can be classified as chemical or natural. Recently, there has been an increased emphasis on the use of spices and aromatic vegetables as food ingredients and natural preservatives. Not only do these ingredients add flavor and aroma to a wide range of foods, but they also possess natural antioxidant properties and contain antimicrobial compounds [67,69].

It is important to keep in mind that preservatives can serve different functions depending on the type of product, ingredients, and processing methods used. In the formulation of PBMA burgers, various ingredients may be added for multiple functional purposes; however, it is crucial to consider the preservative role of these to ensure the quality and safety of the final product.

Onions are ingredients frequently present in the ingredient list of PBMA burgers. These are valued not only for their distinctive flavor and aroma but also for their demonstrated antimicrobial properties. This antimicrobial activity is due to the presence of thiosulfinates and other volatile organic compounds found in onions [69]. On the other hand, salt is a well-known antimicrobial agent that works by reducing water activity through its osmotic effect, and similarly, sugar can act as a preservative by removing excess moisture and inhibiting the growth of microorganisms. In addition to their sensory and functional properties, these ingredients play a crucial role in ensuring the quality and safety of PBMA burgers by preventing spoilage and the growth of harmful microorganisms [72].

Food acids are widely used in the food industry to enhance the flavor of products, as well as to act as preservatives and antioxidants. Some commonly used food acids include vinegar, citric acid, tartaric acid, malic acid, fumaric acid, lactic acid, and sorbic acid [65]. Additionally, certain herbs and spices, such as clove, oregano, thyme, cumin, cinnamon, and rosemary, have been found to improve the shelf life of food products due to their antioxidant, antifungal, and antimicrobial effects. In fact, research has shown that blends of different spices can exhibit even stronger inhibition against specific bacteria than individual spices alone [67].

Several companies offer protective microorganism cultures like *L. carnosum* or *Lb. plantarum*, which is known for its ability to inhibit the growth of spoilage organisms and pathogens in food products. These cultures positively influence the product’s microflora through their unique microbiological and enzymatic properties, ensuring prolonged freshness [66,73].

Additionally, various techniques can be applied to the burger’s ingredients or to the final product to extend its shelf life, such as pre-cooking, cooking, freezing, and frying. Packaging materials and methods are also decisive in preserving the quality and freshness of the burger [48,71]. For instance, vacuum-sealed packaging or modified atmosphere packaging (MAP) with nitrogen or carbon dioxide can reduce the amount of oxygen in the package, slowing down the oxidation process that can cause food spoilage [70]. Finally, post-production preservation characteristics during transport and storage, such as keeping the product refrigerated at a specific temperature, are also determinants to ensure that the product remains fresh and safe for consumption.

### 3.7. Fortification

In the context of PBMAs, fortification is used to address the fact that these products may not contain all of the essential nutrients found in traditional animal-based products [27]. By fortifying the products with essential nutrients, manufacturers aim to make them a more nutritious alternative to traditional meat products. However, this strategy leads to additional ingredient costs and longer ingredient lists, which cannot be well appreciated by consumers [35].

It is important to note that the insufficient intake of nutrients found in animal meats, such as iron, zinc, niacin, riboflavin, vitamin B6, and vitamin B12, is a potential concern with consuming a plant-based diet [74]. Therefore, fortifying PBMA burgers with these nutrients might make them a more attractive option for individuals who are looking to reduce their meat consumption or switch to a vegetarian or an exclusively plant-based vegan diet.

Unlike conventional animal burgers, plant-based burger products often make explicit claims about their nutritional value on their label [17]. Some of the most common nutritional claims regarding fortification in PBMAs are their inclusion of vitamin B12, iron, and zinc [16,75]. PBMA burgers can also be claimed as “high in” or a “source of” protein or fiber. When adding ingredients for fortification purposes, certain factors should be considered. In the European market, fortified ingredient quantity should follow the EFSA criteria in order to make a nutritional claim of a food product [76].

Regarding protein fortification, the final protein content of a food product is primarily determined by the amount of protein added in the form of powder or TVP. The digestibility and amino acid profile depend mainly on the source of the protein and the treatment applied during processing. Nonetheless, the protein’s nutritional claims only give information on the total protein content, and protein quality is not considered [76]. In this scenario, attaining a protein content higher or equivalent to that found in traditional animal-based burger products holds significant importance.

When it comes to including minerals and vitamins in a product, these can either be added as purified individual ingredients or within matrices such as microalgae, mushrooms, or pulse flour. However, it is crucial to consider the stability and bioavailability of both the additives and ingredients [20,77].

For example, the bioavailability of iron varies depending on its form; non-heme iron has low bioavailability, whereas heme-bound iron has high bioavailability [78]. As such, heme-iron from soy leghemoglobin seems promising, which has an equivalent bioavailability to iron from bovine hemoglobin when supplemented in a food matrix, even though further research is needed to fully understand the potential benefits and drawbacks [79]. Generally, iron is introduced into the food matrix of some PBMA products in many different forms, such as iron sodium EDTA, ferrous sulfate, and microencapsulated iron diphosphate, among others [10]. Additionally, it is important to assess the presence of inhibitors and enhancers to ensure the optimal bioavailability of vitamins and minerals. For instance, EDTA and ascorbate are iron bioavailability enhancers [78,80], and these can be seen together with iron in the ingredient list of some PBMA burger products. On the other hand, phytic acid is a potent inhibitor of iron absorption even at low concentrations, and this should be considered [78].

Since vegetarians have limited natural sources of B12 (milk, dairy, and eggs) [81] supplementation through food supplements and fortified food is recommended to prevent deficiency in these populations [82]. There are four authorized forms of vitamin B12 for supplementation purposes: cyanocobalamin (CNCbl), hydroxocobalamin (OHCbl), 5′-deoxyadenosylcobalamin (AdoCbl), and methylcobalamin (MeCbl). All four forms are effective at improving vitamin B12 levels in the human body, as reported in various studies. In accordance with the Commission Regulation (EC) No 1170/2009 [83], CNCbl, OHCbl, AdoCbl, and MeCbl may also be used in the manufacture of food supplements, whereas CNCbl and OHCbl can be added to foods [82,84]. The most commonly used supplemental form found in PBMA products is (CNCbl) due to its relatively low production cost and stability when exposed to heat. Alternatively, (OHCbl) may also be used such as in the form of hydroxocobalamin acetate [79].

Regarding zinc fortification, a range of different forms can be used, including zinc sulfate, zinc oxide, zinc citrate, zinc acetate, and zinc gluconate, among others [85]. These forms of zinc can be added to food products to increase their zinc content, bringing it to levels comparable to those found in animal products such as beef. However, it is worth noting that the bioavailability of zinc from these forms is comparatively lower than that found in animal products [79].

## 4. Clean Label

In recent years, there has been a growing focus on enhancing the quality characteristics of meat-like processed products through technological advancements and innovative formulations. This often involves, as described previously, the incorporation of various additives to replicate the texture, juiciness, mouthfeel, and flavor of meat in order to mimic meat properties. However, such practices have raised concerns about the potential impacts on nutrition, food safety, labeling, production costs, and consumer trust [30]. Additionally, health-conscious consumers pay a lot of attention to the nutritional profile of meat analogues, where the overall nutritional profile of the final product mainly depends on the combination of all the ingredients used. Table 4 gathers some of the main consumer concerns regarding the nutritional and ingredient profile of PBMA products.

Consumers often have a negative reaction to lengthy ingredient lists featuring scientific names or E numbers. This encourages producers to look for ingredients that can be labeled or perceived as natural, such as fibers, natural flavors, colorants, etc. This is essential because the clean label trend is gaining momentum, and there is a growing interest among consumers to learn about the ingredients present in their food [14]. Even though there is no legal definition for the “clean label” term, some definitions and interpretations have been proposed. However, the term “clean label” is typically applied to a food based on whether certain ingredients (such as additives and preservatives) are present or absent in the food [87]. So, to measure the nutritional value perception of PBMAs, the incorporation of natural ingredients and its formulation process plays a crucial role; however, other terms that can raise concerns include the degree of processing. For this, systems like NOVA can help consumers classify food products based on their degree of processing [86]. While some brands rely heavily on processed ingredients that may include genetically modified protein (GMO), more and more manufacturers are opting for non-GMO protein sources and adhering to “clean label” principles [11,28].

Also, there is ongoing speculation and debate about the safety of binders and gums found in PBMAs. One such ingredient is methylcellulose (MC), a modified cellulose dietary fiber commonly used in modern meat analogue products, particularly PBMA burgers. When used in appropriate quantities, MC is a highly effective binder [91]. Some MC alternatives that can be declared as natural fibers in the ingredient list have been patented; however, there is not any ingredient in the market that can replicate the unique properties of this ingredient. Also, the use of various types of gums (such as acacia gum, guar gum, xanthan gum, and others) has sparked controversy over their nutritional impact. The safety and well-being of these products have been questioned, but thus far, no concrete evidence of any health risks or concerns has been found [92].

Some negative concerns are additionally related to the type of fat used. Coconut fat, due to its high amount of medium-chain saturated fatty acids, has generated debate about whether it has either positive or negative effects on health; however, more studies are needed to confirm a further hypothesis [88,89]. Other types of oil, such as sunflower oil, have been in controversy due to the high amount of omega 6. Furthermore, the process should be taken into consideration when evaluating the oil quality.

Also, salt is an important parameter that should be considered. It is well known that consuming salt in high doses can lead to health problems. However, reducing the salt content of the product is not an easy task since it not only influences the flavor perception but also other parameters such as texture or preservation [72]. On the other hand, the total energetic content might also play a role in consumer acceptance since this parameter has been shown to highly influence consumer purchases [90].

Plant-based burgers often contain allergens, such as soy, wheat, peanuts, tree nuts, mustard, and sesame, which can be a concern for individuals with allergies to these ingredients. To address this issue, it is important to consider making allergen-free plant-based burgers. When formulating food products, allergens are typically avoided whenever possible. In the case of PBMA burgers, gluten/wheat is the most commonly declared allergen, followed by soy. As commented, different ingredients with essential functionalities contain these allergens, and this creates a complex balance between ensuring food safety for consumers with allergies and maintaining the desired taste and texture of the product [20].

Some products do contain a front-of-pack label, such as Nutrisocre, that provides user-friendly information regarding its global nutritional profile. According to several studies, PBMA burgers tend to receive more favorable scores than animal-origin burgers [18,19]. Some of the commented ingredients do have a direct impact on the Nustricore punctuation or other front packs. So, formulating the product with the aim of having a positive score is a challenging strategy to increase product acceptance. In this frame, applying clean label ingredients, not only free of E numbers but with high-quality ingredients, can further reinforce the position of meat analogues in the market. This is applicable not only to vegans and vegetarians but also to consumers who are seeking healthier food alternatives [11].

## 5. Perspectives and Future Trends of PBMA Burgers

In recent years, significant progress has been made in the development of PBMA burgers. As mentioned, soy, wheat, and pea burgers based on TVP are currently the primary products of this new generation of PBMA products. Even so, new technologies and novel ingredients are emerging.

Ongoing research explores diverse methods for protein concentration and hydrolyzation because a wide range of functionalities can be achieved [33]. Innovation is applied to other protein texturization methodologies rather than high-moisture extrusion or low-moisture extrusion [22,31]. New emerging technologies to create PBMAs are gaining popularity, such as shear cell technology, which allows anisotropic structures to serve as meat replacers [93]. These technologies include electrospinning, which can produce fibrous materials from proteins like zein [94]; wet spinning, which has the capacity to produce food-grade fibers from soy, pea and fababean [95]; freeze structuring, which can produce fibrous and layered structures from plant proteins such as pea and wheat [96,97]; and 3D printing, a technology that is widely used by different companies to create PMBAs through precise control of plant protein addition through a robotic-process [98].

Regarding plant-based ingredients, different protein sources are gaining popularity. Different lines of research are focused on the use of alternative ingredients to partially or totally substitute soy, pea, or wheat proteins. Algae are particular ingredients in the scope of interest. As reported by Grahl et al. [99], different types of algae in combination with soy were shown to create fibrillary textured extrudates. The same happened with hemp protein combined with soy, where Zahari et al. [55] showed the different possibilities to produce HMMAs. Other proteins, such as zein, have offered promising results due to their unique properties to produce fibration [95]. Furthermore, some authors have reported that jackfruit is a potential and versatile ingredient to mimic meat burger properties due to its fibrous texture and mild flavor [100].

Lastly, it is important to mention exist other alternatives exist beyond plant-based meat analogue burgers. There are non-plant-based meat analogue options that have offered remarkable results. Lab-based meat is a very promising technique, which consists of culturing animal cells in a laboratory. However, the progress of lab-based meat is in its initial phase, encountering notable obstacles with significant challenges, such as technological limitations and consumer acceptance [101]. Another emergent topic is insect protein, which is offered as a promising new source of protein for human food in the future [102], but also consumer acceptance of insect-based meat remains a challenge. Additionally, some mycoproteins, derived from fungi, can be used as meat analogues due to their ability to mimic the taste, texture, and nutritional profile of meat products, offering a sustainable and allergen-friendly alternative food product [103]. Other products that are emerging as an alternative to traditional meat products are hybrid meat products, which are produced using a combination of plant and animal ingredients in varying proportions. These products have the potential advantage of providing a familiar meaty taste and texture along with the nutritional and environmental advantages derived from plant sources [104].

## 6. Conclusions

The development of plant-based meat analogue (PBMA) burgers heavily relies on the functional attributes of various ingredients, including proteins, binding agents, texturizers, fats, flavoring agents, coloring agents, and preservatives, which are meticulously selected and combined to replicate the taste, texture, and nutritional content of traditional meat products while fortifying PBMA’s nutritional profiles. As technology advances and consumer demands evolve, continued innovation in formulation, fortification, and clean label strategies is essential for driving the growth and acceptance of plant-based meat analogues globally. However, creating PBMA burgers with simpler, natural ingredients and clean label status while maintaining nutritional profile and taste presents a significant challenge in the industry. Recent advancements primarily utilizing soy, wheat, or pea proteins, alongside ongoing research exploring new protein concentration and texturization methods, have shown promise. Yet, alternative ingredients like lab-grown meat, insect protein, mycoproteins, and hybrid meat products offer additional opportunities, each with its own set of challenges. Embracing this diverse range of innovations is pivotal to meeting the growing demand for sustainable protein sources and shaping the future of food, though their success hinges on overcoming scalability and economic viability issues, emphasizing the need to ensure sustainable production methods and affordability for consumers.

## Figures and Tables

**Table 1:** Scientific paper reviews describing plant-based meat analogue ingredients and their functionalities.

Study Type	PBMA Product Type	Described Ingredients	Reference
Review article	general	Fats, thickening agents, adhering agents, colorants, flavorings, minerals, vitamins, antioxidants, antimicrobials	[10]
Review article	general	Nonanimal proteins, lipids, polysaccharides, flavoring ingredients, coloring agents, fortification ingredients	[11]
Review article	general	Protein ingredients, lipid ingredients, carbohydrate ingredients, flavor enhancers, coloring agents	[21]
Book section	general	Proteins, fat or oil, binding agents, flavorings and taste enhancers, coloring agents	[24]
Review article	general, emulsion-type, burgers/patties/nuggets, chickens/steak	Plant proteins, binding and texturizing agents, fat, oil and oil substitutes, flavor and coloring agents, water	[29]
Review article	general	Proteins, coloring ingredients, flavoring ingredients	[28]
Review article	general	Proteins, oil and fats, binding agents, taste and flavor enhancers, coloring agents	[30]
Systematic literature review article	general	Texturized vegetable proteins, binding agents, fat/oil, and other ingredients	[31]
Review article	general	Plant proteins, coloring agents, flavors, and other ingredients	[32]

**Table 2:** Main functionality and source of the macronutrients of PBMA burgers.

Ingredient	Source	Main Functionality	Reference
Carbohydrates (polysaccharides)	Starches, flours, fibers, and purified polysaccharides	Thickening, emulsification, water and oil retention and gelation	[10,11,29]
Sugars	Sucrose, dextrose, maltose, xylose etc.	Flavor and color “Maillard reaction”.	[38,39]
Fats	Low saturated fatty acid oils: (e.g., sunflower oil, olive oil, corn oil, turnip oil, and canola oil) and high saturated fatty acid oils: (e.g., coconut oil, palm oil, and cocoa butter)	Texture contribution (tenderness, mouthfeel, juiciness) and flavor release.	[12,34,40]
Proteins	Texturized, isolates and concentrates: (e.g., soy, wheat, pea, chickpea, faba bean, rice, and sunflower)	Texturization, mouthfeel and texture contribution, emulsification, oil and water retention, flavor binding, nutritional value	[29,30,41]

**Table 3:** Principal methods to extend the shelf life of PBMA burgers.

Method	Source/Type	Specific Methodology	Reference
Addition of preservatives	Natural ingredients	Spices and aromatic vegetables	[66,67,68,69]
	Processed ingredients	Acids, sugar, salt	
	Microorganism culture	*L. carnosum, Lb. plantarum*	
Processing treatment	High temperature	Pre-cooking, cooking, frying	[48]
	Low temperature	freezing	
Packaging	Air-removal	Vacuum sealing	[69,70]
	Air replacement	Modified atmosphere (nitrogen, carbon dioxide, etc.)	
Preservation/transport	Low temperature	Refrigeration, freezing	[48,71]

**Table 4:** Clean label consumer concerns.

Concern Problem	Proposed Solution	Reference
Lengthy ingredient list	NOVA system	[86]
E-numbers	Substitution of the E-numbers for “natural ingredients”	[87]
Genetically modified ingredients (GMO)	Use alternative ingredients produced by non-GMO practices	[11,28]
Elevated saturated fat or omega 6 fatty acids	Reduce or substitute the fat with high quality oils rich in omega 3 (e.g., olive oil)	[88,89]
High salt content	Lower the salt addition	[72]
Elevated energy content	Reduce energetic content by fat reduction or addition of fiber	[90]
Presence of allergens	Production free allergen products	[20]

## Data Availability

Not applicable.
